# Nox4 Maintains Blood Pressure during Low Sodium Diet

**DOI:** 10.3390/antiox10071103

**Published:** 2021-07-10

**Authors:** Flávia Rezende, Pedro Felipe Malacarne, Niklas Müller, Birgit Rathkolb, Martin Hrabě de Angelis, Katrin Schröder, Ralf P Brandes

**Affiliations:** 1 Institute for Cardiovascular Physiology, Goethe University, Theodor-Stern Kai 7, 60590 Frankfurt, Germany; malacarne@vrc.uni-frankfurt.de (P.F.M.); nmueller@vrc.uni-frankfurt.de (N.M.); schroeder@vrc.uni-frankfurt.de (K.S.); brandes@vrc.uni-frankfurt.de (R.P.B.); 2 German Center of Cardiovascular Research (DZHK), Partner Site Rhein Main, 60590 Frankfurt, Germany; 3 Institute of Experimental Genetics, German Mouse Clinic, Helmholtz Zentrum München, German Research Center for Environmental Health (GmbH), Ingolstaedter Landstr. 1, 85764 Neuherberg, Germany; birgit.rathkolb@helmholtz-muenchen.de (B.R.); hrabe@helmholtz-muenchen.de (M.H.d.A.); 4 Institute of Molecular Animal Breeding and Biotechnology, Gene Center, Ludwig-Maximilians University, Feodor-Lynen Str. 25, 81377 Munich, Germany; 5 German Center for Diabetes Research (DZD), Ingolstaedter Landstr. 1, 85764 Neuherberg, Germany; 6 Experimental Genetics, TUM School of Life Sciences, Technische Universität München, Alte Akademie 8, 85354 Freising, Germany

**Keywords:** NADPH oxidase 4, proximal tubule cells, reactive oxygen species

## Abstract

The NADPH oxidase Nox4 is a hydrogen peroxide (H_2_O_2_)-producing enzyme, with the highest expression in the kidney. As the kidney is involved in volume and blood pressure control through sodium handling, we set out to determine the impact of a low sodium diet on these parameters in WT and Nox4-/- mice. Nox4 expression in the murine kidney was restricted to the proximal tubule. Nevertheless, low-sodium-induced weight loss and sodium sparing function was similar in WT and Nox4-/- mice, disputing an important function of renal Nox4 in sodium handling. In contrast, a low sodium diet resulted in a reduction in systolic blood pressure in Nox4-/- as compared to WT mice. This was associated with a selectively lower pressure to heart-rate ratio, as well as heart to body weight ratio. In general, a low sodium diet leads to activation of sympathetic tone and the renin angiotensin system, which subsequently increases peripheral resistance. Our observations suggest that the control by this system is attenuated in Nox4-/- mice, resulting in lower blood pressure in response to low sodium.

## 1. Introduction

Reactive oxygen species (ROS) and oxidative stress have been implicated in kidney disease [1]. Numerous ROS generator systems are present in the kidney and have been linked to renal pathologies. An important ROS generator system is the Nox family of NADPH oxidases (Noxes), which are expressed in the kidney and may contribute to ROS-dependent pathologies [2,3,4,5].

The individual cells of the kidney exhibit a cell-specific expression pattern of the different Nox homologues, as well as a differential response to Nox enzyme-inducing and -activating stimuli. In models of angiotensin II infusion and a high salt diet, the renal expression of Nox2 and its accessory subunits is increased [3] and the hypertension induced in response to these stimuli has been, at least in part, attributed to an increased renal production of superoxide anions and/or hydrogen peroxide [6,7,8]. Despite a growing body of literature, the specific functions of most NADPH oxidases in the kidney are insufficiently understood. This aspect is particularly true for the NADPH oxidase Nox4. In fact, Nox4 was initially identified in the kidney. At the protein level, the kidney has, by far, the highest Nox4 expression. Conversely, in all other organs Nox4 is hardly detectable; the kidney yields a strong and specific signal for Nox4 by Western blot [9]. Different to all other Nox enzymes, Nox4 is constitutively active and is only dependent on p22phox [10], not on cytosolic subunits. Moreover, Nox4, like the Duox-NADPH oxidases, directly produces H_2_O_2_, due to its ability to trap O_2_^•-^ in a pocket of its E-loop [11]. H_2_O_2_ is a relatively long-lived ROS, which can exert a signaling function through direct reaction with cysteines [12] and the metal centers of enzymes [13]. Despite numerous publications [14,15,16], there is currently no consensus regarding the role of Nox4 in renal disease, as interpretations vary according to the loss of function strategy (inhibitor, knockdown, knockout, dominant negative enzyme) or model system (cell culture, mouse model, rat model) [17,18,19,20,21]. In models of diabetes and renal fibrosis, knockout of Nox4 did not result in renal protection [9,22]. In contrast, in hypertension and kidney injury in a Dahl salt-sensitive (SS) rat model, knockout of Nox4 attenuates blood pressure increase in response to a high salt diet (4%) [23]. Given that the sodium-sparing function of the kidney is essential to body water conservation and, thus, blood pressure maintenance through plasma volume control, the function of Nox4 in this context becomes an important question. We therefore hypothesized that Nox4 contributes to renal sodium handling, and studied this aspect using a low sodium diet in wild-type (WT) and Nox4-/- mice.

## 2. Material and Methods

### 2.1. Knockout Animals and Animal Procedure

Tamoxifen-inducible Nox4-/- mice (Nox4flox/flox-ERT2-Cre+/0 mice) were generated by crossing Nox4flox/flox mice (backcrossed more than 10 generations into C57/Bl6J) with CreERT2+/0 mice, as described previously [24]. Knockout was induced by tamoxifen chow (LasCRdiet CreActive TAM400 (400 mg/kg) for 10 days followed by a wash out time of 14 days. A low sodium diet (0.01% Na^+^) was obtained from Altromin (#C1036). Urine was collected in metabolic cages, and blood was collected in lithium heparin tubes. Plasma was obtained by 2000× *g* centrifugation for 5 min at 4 °C.

All experiments performed with animals were in accordance with German animal protection laws and were carried out after approval by the local authorities under the number FU1089. Animals were housed in groups with free access to chow and water in a specified pathogen-free facility with a 12/12 h day/night cycle. Given the impact of gender on ROS production, only male animals older than 8 weeks were used in this study.

### 2.2. RNAscope^®^ In Situ Hybridization Combined with Immunofluorescence

RNAscope in situ hybridization and protein detection by immunofluorescence were performed on paraffin embedded renal sections. Adult murine kidneys were fixed in 4% paraformaldehyde at room temperature overnight, dehydrated, then embedded in paraffin and cut into 5 µm thick sections. RNAscope was performed according to the manufacturer’s protocol (Advanced Cell Diagnostics, Hayward, CA, USA). Briefly, tissue sections were heated for 1 h at 60 °C and, subsequently, deparaffinized in xylene (2 × 5 min), isopropanol (2 × 5 min), ethanol (2 × 5 min) and then air-dried for 5 min. Samples were pretreated with hydrogen peroxide for 10 min and then with a target retrieval solution followed by Protease Plus reagent for 30 min at 40 °C. After Protease Plus, slides were washed in ddH_2_O and incubated with Nox4 probe for 2 h at 40 °C. Peptidylprolyl isomerase and *B. subtilis* dihydrodipicolinate reductase were used as a positive and negative control, respectively. Following probe hybridization, the RNAscope 2.0 HD Detection Kit–Brown (for detection of onlye RNA) or –Red (to combine with immunofluorescence) was applied for visualizing hybridization signals.

For immunofluorescence staining, slides were washed in PBS after performing RNAscope. Subsequently, specimens were blocked with a NaN_3_ solution for 1 h at 37 °C. After washing in PBS, probes were blocked a second time using a milk solution for 20 min at room temperature, and then incubated with primary antibodies (1:500 diluted in Roti) overnight at 4 °C. The primary antibodies used for immunofluorescence were anti-aquaporin 2 (AQP2) (C-17; Santa Cruz Biotechnology) and anti-megalin (Meg) (P-20; Santa Cruz Biotechnology). The slides were then washed in PBS and sections were incubated with the secondary antibody for 2 h at room temperature. Secondary antibody (1:500 diluted in Roti) was Alexa Fluor 488 goat anti-rabbit IgG (A11034; Invitrogen). Slides were washed in PBS then treated with DAPI (1:500 diluted in PBS) and finally covered using Mounting Medium (P36931; Thermo Fisher). RNAscope in situ hybridization and immunofluorescence images were captured on a confocal microscope (LSM800; Carl Zeiss, Germany).

### 2.3. Amplex Red Measurements from Renal Tissue

The kidneys were freshly isolated, separated into cortex and medulla, and HT buffer (buffer containing in mmol/L: 137 NaCl, 2.7 KCl, 0.5 MgCl_2_, 1.8 CaCl_2_, 5 glucose, 0.36 NaH_2_PO_4_, 10 HEPES) was added to 1 mL/mg tissue. The organs were then minced with a scalpel and H_2_O_2_-derived fluorescence was measured in the supernatant of 100 mg tissue in the presence of Amplex Red (50 μmol/L, Invitrogen) and horseradish peroxidase (2 U/mL). Fluorescence was determined in the supernatant at 540 nm/580 nm excitation/emission. H_2_O_2_ concentration was estimated from a standard curve.

### 2.4. Blood Pressure Measurements

Tail cuff measurements were performed with a 6–channel setup (Vistech BP2000). Measurements over 5 days were averaged per mouse.

### 2.5. Clinical Chemistry

Electrolytes (Na^+^, Cl^−^, K^+^) in plasma and urine were analyzed with an AU480 clinical chemistry analyzer (Beckman Coulter Germany, Krefeld, Germany) with the integrated ion-selective electrodes unit (ISE) and reagent kits provided by Beckman Coulter, according to previous publications [25]. Creatinine was measured by an enzymatic method using a kit from Beckman Coulter (#OSR61204).

Clearance was calculated by dividing the excretion rate (concentration in urine multiplied by urine flow) by plasma concentration.

### 2.6. Statistics

Unless otherwise indicated, data are given as means ± standard error of mean (SEM). Calculations were performed with Prism 5.0 or BiAS.10.12. The latter was also used to test for normal distribution and similarity of variance. In the case of multiple testing, Bonferroni correction was applied. For multiple group comparisons, ANOVA variance testing followed by post hoc testing was performed. Individual statistics of unpaired samples was performed by a t-test, and if found to be not normal, distributed by the Mann–Whitney test. A pvalue of <0.05 was considered as significant. Unless otherwise indicated, n indicates the number of individual experiments or animals.

## 3. Results

### 3.1. Nox4 Expression Is Restricted to Proximal Tubule Cells

**Figure 1:**
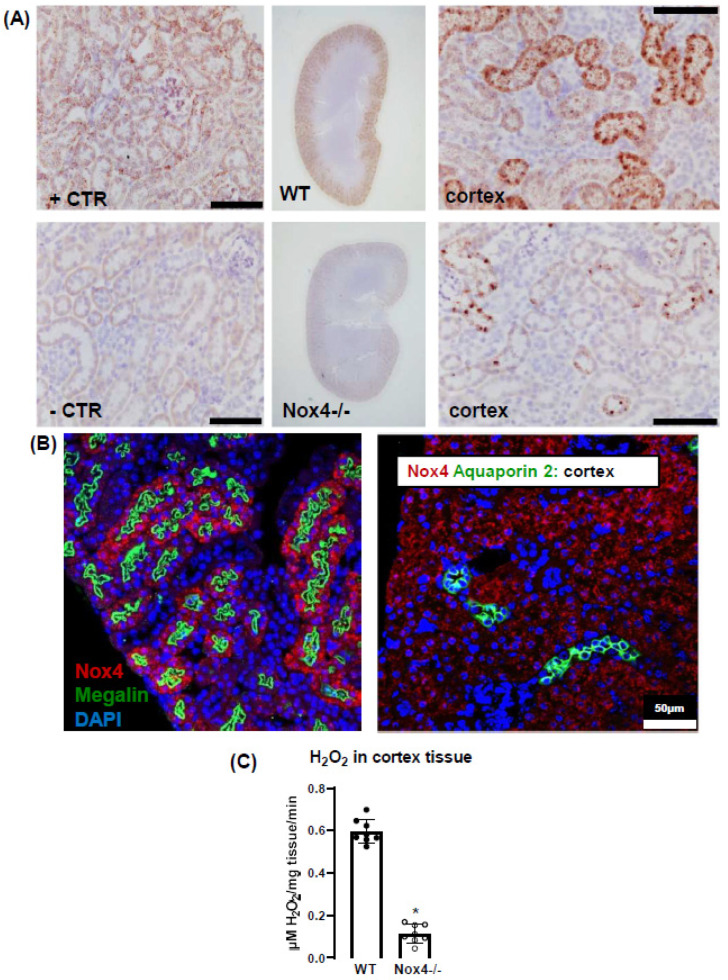
Nox4 is expressed in the proximal tubule cells and produces H_2_O_2_. (**A**): RNAscope in kidney. Left panel: +CTR: peptidylprolyl isomerase (**B**), −CTR: *B. subtilis* dihydrodipicolinate reductase. Middle: staining of Nox4 in cortex and medulla of WT and Nox4-/- mice. Right panel: higher magnification. Scale bar: 20 µm. B: RNAscope combined to IF shows Nox4 expression at the proximal tubule and not at the collecting tubule. (**C**): H_2_O_2_ measurement from renal tissue using Amplex red. * *p* < 0.05. *n* = 8 mice for each group.

To begin, we determined which cells of the kidney express Nox4. For this, we used high-resolution in situ hybridization (RNAscope^®^) to visualize Nox4 mRNA, which has been reported to strongly correlate with the protein level of Nox4 [26]. A custom-designed probe and chromogenic staining (Brown kit) confirmed that Nox4 mRNA expression is restricted to the renal cortex (Figure 1A). No staining was detected in the kidneys of Nox4-/- mice. To specifically identify the Nox4 mRNA expressing cell, we combined RNAscope^®^ with immunofluorescence using megalin and aquaporin-2 as markers for proximal tubule and collecting duct cells, respectively. Nox4 staining was restricted to megalin-positive cells (Figure 1B); also in addition, no staining was observed in the glomeruli (visualized by its distinct morphology). These results demonstrated that Nox4 expression in the kidney is restricted to the proximal tubule cells.

### 3.2. Nox4 Contributes to H_2_O_2_ Production of the Renal Cortex

Next, we measured H_2_O_2_ as a readout for an active Nox4 enzyme. For this, we utilized Amplex red^®^ with HRP as a sensitive method for H_2_O_2_.

Freshly dissected kidneys from WT and Nox4-/- mice were separated into cortex and medulla and minced in HT (Hepes Tyrode) buffer and incubated with Amplex red^®^/HRP solution. Renal cortex of Nox4-/- showed a significantly lower (16% of WT mice) release of H_2_O_2,_ demonstrating that Nox4 is the source of H_2_O_2_ in the murine renal cortex (Figure 1C).

### 3.3. Knockout of Nox4 Lowers Blood Pressure and Cardiac Mass in Response to Low Sodium Diet

The proximal tubule is the renal site of mass absorption. More than 65% of the water, a high proportion of bicarbonate and phosphate, and basically all amino acids, glucose, as well as numerous vitamins and trace elements, are recycled at this site. Almost all transport processes in the proximal tubule are coupled, directly or indirectly, to sodium reabsorption. The fine-tuning of sodium excretion occurs in the late distal tubule and collecting duct. A previous publication on the role of Nox4 in salt-induced hypertension in Dahl rats [27] suggests that Nox4 has an impact on sodium handling and, thus, total body water content and plasma volume.

**Figure 2:**
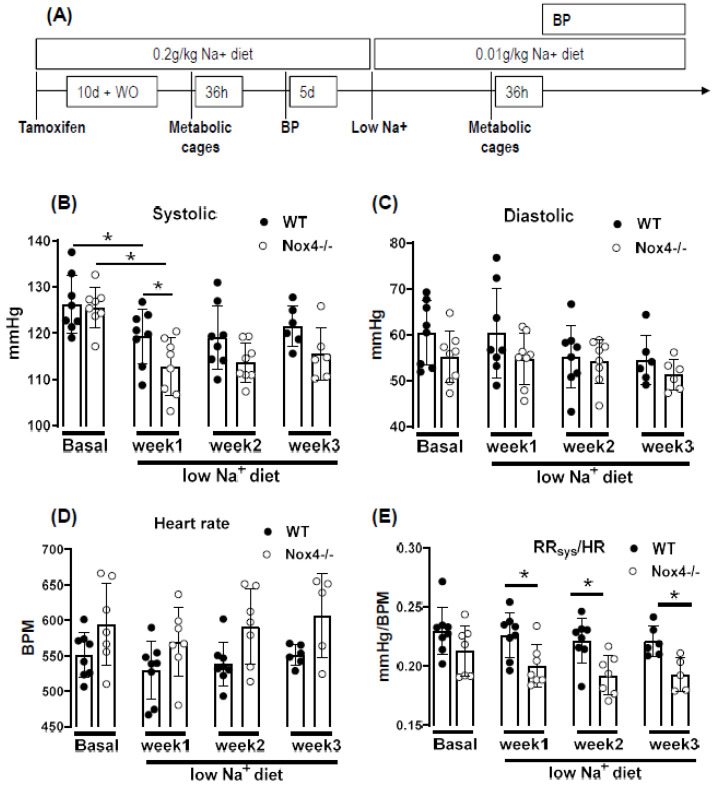

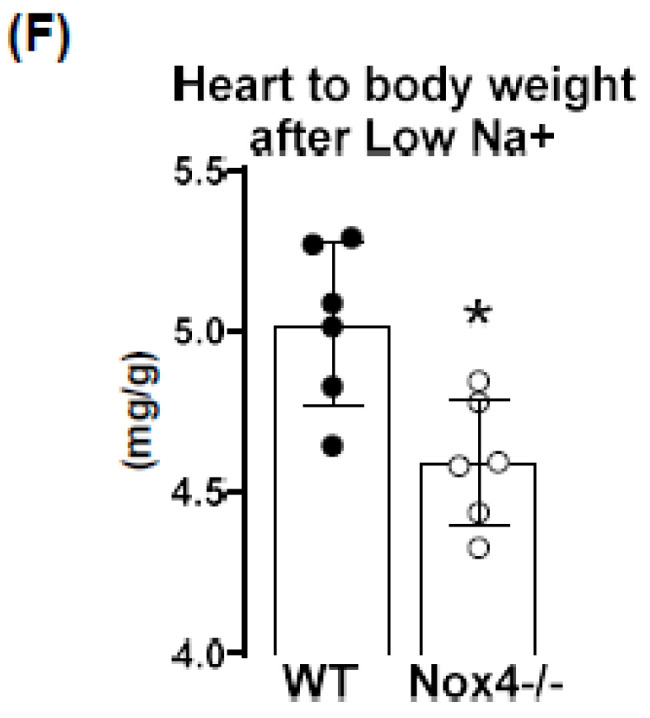
Experimental setup and cardiovascular parameters. (**A**): experimental design. (**B**): systolic blood pressure. (**C**): diastolic. (**D**): heart rate. (**E**): ratio of systolic blood pressure to heart rate. (**F**): ratio of heart to body weight. * *p* < 0.05, non-parametric test, Mann–Whitney. *n* ≥ 6.

To study this aspect, mice were challenged with a low sodium diet: body weight, blood pressure and sodium excretion were first studied on regular chow (0.2 g/kg sodium), and subsequently on a low sodium diet (0.01 g/kg) applied for up to three weeks with prior tamoxifen-mediated knockout of Nox4 (Figure 2A).

Under normal chow, the blood pressure and heart rate of WT and Nox4-/- mice were similar. A reduction in sodium intake slightly lowered systolic blood pressure in both strains; this effect was more pronounced in Nox4-/- than in WT mice (only significant at week 1 of low Na+). There was also a trend towards a higher heart rate in Nox4-/- vs. WT mice (Figure 2B–D). The values do not reach significance at all timepoints, potentially due to insufficient group size or high variability in tail cuff measurements. The combination of lower blood pressure and increased heart rate might indicate that peripheral resistance or plasma volume are different between WT and Nox4-/- mice. Indeed, whereas there was no difference under normal chow, when exposed to a low sodium diet, the ratio of systolic blood pressure to heart rate was significantly lower in Nox4-/- compared to WT mice (Figure 2E). Lower peripheral resistance or volume should attenuate cardiac load (after and preload, respectively), thus resulting in small hearts. Indeed, at the end of the 3-week diet, the heart to body weight ratio was lower in Nox4-/- as compared to WT mice, whereas the body weight was similar (Figure 2F).

### 3.4. Knockout of Nox4 Does Not Alter Body Weight and Water Intake in Response to Low Sodium Diet

**Figure 3:**
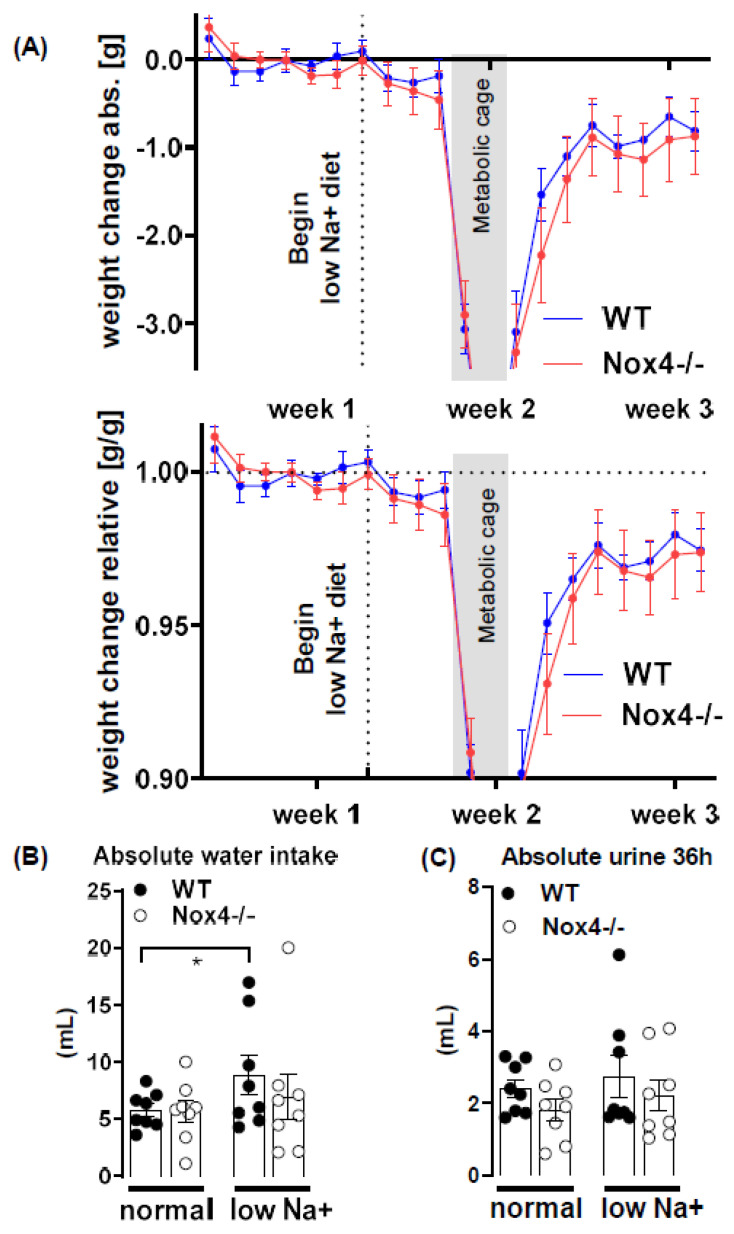
Effect of diet on weight, water intake und urine excretion. (**A**): absolute and relative weight change during the experiment. The grey box indicates the time mice were kept in metabolic cages. Vertical dotted line indicates the beginning of a low sodium diet. (**B**,**C**): Absolute water intake and urine production determined in the metabolic cage before and 3 days after the initiation of a low sodium diet. (36 h collection time) *n* = 8. * *p* < 0.05 paired Wilcoxon test, data are mean ± SEM.

A reduction in food sodium content resulted in a loss of body weight of approx. 1 g within 2 weeks. Although this weight loss was, on average, slightly greater for Nox4-/- mice at any timepoint, the difference did not reach the level of significance (Figure 3A). As determined by metabolic cages performed prior to and 3 days after the initiation of the low sodium diet, the diet slightly increased water intake, which reached the significant level for WT mice but not for Nox4-/- mice (paired test, Figure 3B). Urine production was similar between all groups (Figure 3C). Thus, there are no profound differences in body water control between WT and Nox4-/- mice.

### 3.5. Knockout of Nox4 Does Not Affect Renal Clearance nor Plasma Level of Na^+^, Cl^−^ and K^+^

**Figure 4:**
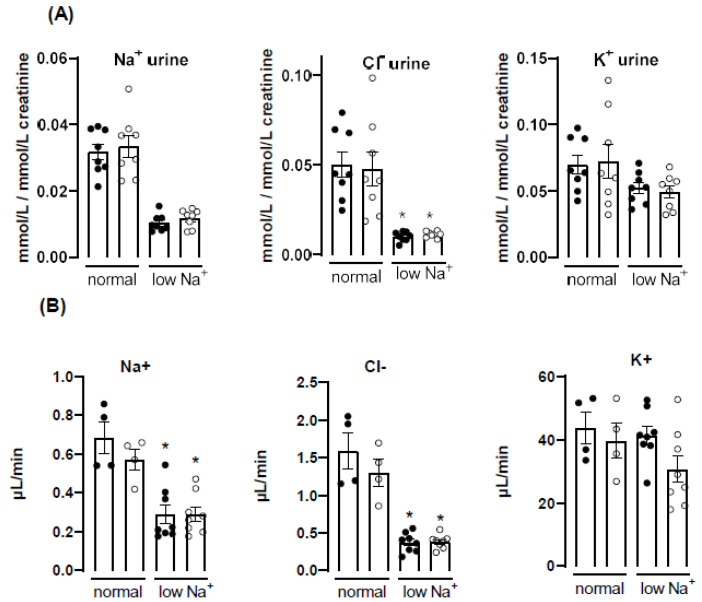
Electrolyte excretion and clearance under normal and low Na+ diet. (**A**): excretion of Na^+^, Cl^−^ and K^+^, normalized by urine creatinine. (**B**): clearance of Na^+^, Cl^−^ and K^+^. * *p* < 0.05, non-parametric test, Mann–Whitney, normal vs. low Na+ diet. Data are mean ± SEM. *n* ≥ 4.

In order to directly determine the role of Nox4 for renal electrolyte handling, plasma and urine electrolytes were measured and their renal excretion and clearance was determined (Figure 4). Sodium restriction resulted in the expected sodium and chloride sparing of the kidney: sodium and chloride excretion, as well as clearance, decreased, whereas the values for potassium remained unchanged. Importantly, there was no difference regarding these parameters between WT and Nox4-/- mice under basal conditions, as well as on a low sodium diet.

## 4. Discussion

In this study, we report that a low sodium diet results in an acute reduction in systolic blood pressure and a prolonged reduction in peripheral resistance in Nox4-/- mice. Despite the high expression of Nox4 in the kidney, this effect was unrelated to salt and water intake and renal sodium handling. Sodium restriction resulted in weight loss and renal sodium sparing, and this effect was similar between WT and Nox4-/- mice. Collectively, these data support the previous report of Nox4 in renal blood pressure control [27] but exclude a direct effect on sodium handling as an underlying mechanism.

In the present study, we observe a high expression of Nox4 in the proximal renal tubule. Our data are in line with the single-cell sequencing atlas of the murine kidney [28], where Nox4 is expressed in the cluster of proximal tubule cells and a novel cluster that is also positive for megalin. In addition, in the human kidney, Nox4 appears to localize to the proximal tubule [18]. Despite these data on expression, very little is known concerning the function of Nox4 in the kidney. The location in the proximal tubule makes studying the function of Nox4 difficult, given that the physiological function of these cells depends on directionality, which is challenging in model systems of isolated cultured cells. Our past data indicated that Nox4 expression in the kidney is highest under quiescent conditions in healthy animals, whereas inflammation and diseases such as diabetes decreased Nox4 [9]. The finding that renal disease reduces Nox4 expression has, meanwhile, been recapitulated by others [18,29] and suggests that Nox4 is a marker for healthy, differentiated, intact renal tissue. This behavior also has significance for renal cell culture models: the isolation of proximal tubule cells leads to a rapid loss in Nox4. Therefore, renal proximal tubule cell lines are alternatives. The opossum kidney OK cell line (from *Didelphis virginiana*) is a broadly used model to study ion transport and membrane trafficking mechanisms in the proximal tubule. Transcriptomics of these cells, compared with mammalian proximal tubule cells, however, reveal that Nox4 expression is also lost in this cultured cell line [30].

Therefore, the search for a function of Nox4 has to rely on in vivo data. Given that the transport processes in the proximal tubule are largely sodium-coupled, studying sodium handling and its indirect consequence, plasma volume and blood pressure, can be seen as first approaches to dissect the function of Nox4 in the kidney. The proximal tubule reabsorbs two-thirds of filtered Na+ [31] and, consequently, is essential in sodium homeostasis. It is also important to note that, despite this behavior, the contribution of this renal segment to volume- and blood-pressure-control is, at least, controversial. From the knowledge available to date, the transport processes in this renal segment are not well controlled, potentially with the exception of phosphate reabsorption, which is inhibited by parathormone. Tubular-glomerular feedback (TGF), which controls proximal tubular urine flux, has a sensor in the distal tubule and affects glomerular filtration rate. To date, TGF has not been linked to Nox4; comparatively, it is associated with nitric oxide and adenosine [32]. The second system that is relevant in conjunction with low sodium is the renin–angiotensin system (RAS) because a low sodium diet increases RAS activity by volume depletion and subsequent sympathetic nerve activation. The combination of increased sympathetic tone and RAS activation usually compensates for the effect of hypovolemia on blood pressure: cardiac output, renal water retention and peripheral resistance all increase. The fact that the blood pressure/heart rate ratio of Nox4 mice on a low sodium diet was significantly lower than that in wild-type mice, suggests that neither peripheral resistance nor volume retention can be adequately increased after deletion of Nox4. As body weight and sodium excretion were similar between WT and Nox4-/- mice, differences in peripheral resistance should be considered.

Interestingly, cerebral knockdown of Nox4 resulted in attenuated sympathico-excitation in response to cardiac damage [33]. Whether Nox4 impacts directly on peripheral resistance is unclear. It has been suggested that SERCA is oxidized by Nox4 in the heart and endothelium [34,35] and also in TRP-channels [36,37,38,39], which contribute to the control of resting calcium, and are targets of Nox4. Moreover, Nox4 has been linked to smooth muscle cell differentiation and, thus, contractility [40,41,42,43]. Collectively, these observations provide support for a role of Nox4 in vascular tone control. Additional studies will be needed to substantiate this assumption.

Is there any evidence for a direct function of reactive oxygen species for renal hypertension? Superoxide anions lead to reduced medullary blood flow and increased sodium retention and, thus, hypertension [7], as shown by in vivo treatment with a superoxide dismutase mimetic. Moreover, superoxide inhibits proximal tubule fluid reabsorption in spontaneously hypertensive rats [44]. Renal hemodynamic and excretory functions, such as urine flow, sodium excretion and glomerular filtration were increased in hypertensive rats infused with the superoxide scavenger tempol without altering arterial pressure [8]. In contrast, infusion of H_2_O_2_ directly into the renal medulla increases mean arterial pressure [6]. Nox4 has been linked to renal hypertension and sodium retention in Dahl salt-sensitive rats, where volume expansion is considered to be the main cause of salt-sensitive hypertension [45]. Our results corroborate the findings in the Dahl salt-sensitive rats; however, we cannot link the effect of Nox4 to Na^+^ homeostasis because Na^+^ excretion and clearance were similar in WT and Nox4-/- mice. On the other hand, the effects might have been so subtle and transient that our study was not sensitive enough to detect them.

The current study has several limitations. Blood pressure was measured by tail cuff technology, which is less accurate than telemetry. Moreover, we only estimated cardiac output and peripheral resistance from the blood pressure to heart rate ratio. A true determination of cardiac output and peripheral resistance would have required indicator injection dilution methodology. Moreover, metabolic cages impose a considerable amount of stress on mice. Food and fluid intake in the cages is low, which is also documented in the present study by the substantial weight loss. A possible alternative would have been clearance measurements using radioactive isotopes, but this technology was not available to us.

## 5. Conclusions

The present study demonstrates that the deletion of Nox4 potentiates lower arterial blood pressure in response to a low sodium diet in mice. The present data may suggest that this is a consequence of a change in peripheral resistance, rather than altered renal sodium handling in Nox4-/- mice.

## Data Availability

Data is contained within the article.
